# Editorial: Mechanical Signaling in Plants: From Perception to Consequences for Growth and Morphogenesis (Thigmomorphogenesis) and Ecological Significance

**DOI:** 10.3389/fpls.2016.01441

**Published:** 2016-10-06

**Authors:** Catherine Coutand, Stephen J. Mitchell

**Affiliations:** ^1^Institut Nationale de la Recherche Agronomique, UMR 547 PIAFClermont-Ferrand, France; ^2^Clermont Université, Université Blaise Pascal, UMR 547 PIAFClermont-Ferrand, France; ^3^Faculty of Forestry, Department of Forest and Conservation Sciences, University of British ColumbiaVancouver, BC, Canada

**Keywords:** thigmomorphogenesis, acclimation to mechanical stress, model of mechanoperception, stretch activated channels, aquatic organisms, mechanosensing in plants

Plant morphogenesis and its regulation have fascinated researchers for more than two centuries. Among determinants of morphogenesis mechanical signals appear as an important cue. The fact that plants respond to mechanical stimuli was reported by Darwin in the 1850's. As described by Iida in this research topic, mechanical stimuli were used in traditional agriculture practices like *mugifumi*. In the past 40 years, the study of mechanical signaling in plants has regained interest because of its implication in fundamental processes of organo- and morphogenesis and their potential as an innovative means of controlling plant growth. The focus of this research topic is the quantification of mechanical signals and of their effects on plant growth, the ecological significance of mechanoperception and thigmomorphogenesis, and the potential use of mechanical stimuli in agriculture practices. The papers in this research topic summarize the current state of knowledge, present new experimental results, identify areas where further investigation is warranted, and propose investigative protocols.

Mechanical signals can come from internal forces due to turgor pressure or externally from the environment. In natural conditions, aerial portions of plants experience mechanical stimuli due to wind, snow and rain, while aquatic plants experience water flow, waves or tides. Plants perceive, transduce respond physiologically to mechanical signals, collectively referred to as thigmomorphogenesis. Thigmomorphogenesis has been described in herbaceous as well as woody plant species. In growing shoots and roots, the first response observed after a mechanical signal is perceived is a transitory growth cessation followed by a progressive recovery of elongation rate and an increase of radial growth rate. When repeated mechanical signals are applied, the classical thigmomorphogenetic growth response of aerial plants is a reduced elongation of axes and, for plants which exhibit an active cambial growth, an increase of growth in girth. Thigmomorphogenesis in aquatic plants has been less well examined.

This research topic includes two articles on mechanosensing in aquatic plants. Schoenlynck et al. examine the response of the aquatic plant *Egeria densa* to hydrodynamic stress and describe the variations of biogenic silicate, cellulose and lignin content and resulting plant mechanical strength. Tesson and Charrier review the role of mechanical signals in brown algae morphogenesis and discuss the advantages of atomic force microscopy to quantify the mechanical signals and the resulting modifications of composition and strength of algal filaments.

For the past 20 years, progress has been made in understanding the mechanosensing signal perception from two points of view: (i) by identifying the sensors; and, (ii) by quantifying the mechanical signals and linking them to responses. In this research topic Peyronnet et al. focus on the role of mechanosensitive protein channels. Moulia et al. review the quantification of mechanical signals perceived by plants and explain how the S3m model of mechanoperception quantifies the link between perception and responses. They illustrate this approach with two examples, the thigmomorphogenetic syndrome of plant shoots bending, and the mechanosensitive control of shoot apical meristem morphogenesis.

The transduction phase refers to the cascade of events within the plants tissues that lead to responses. In Arabidopsis, the expression of more than a thousand genes is modified after signal perception. Among these mechanosensing actors, some are also triggered by heat, salinity and drought. Other actors are far more specific to mechanical stimuli, including touch-genes or the zinc-finger transcription factor PtaZFP2 cloned in poplar. In their review paper, Leblanc-Fournier et al. note that the expression of PTaZFP2 is related to the number and intensity of strain signals, making it an interesting marker in mechanosensing studies. It also appears as a key factor in plant acclimation to recurrent mechanical signals. Also in this research topic, Cazzonelli et al. describe the role of the chromatin modifying enzyme SDG8, which appears as a key enzyme in the mechanosensing process in Arabidopsis by enabling the expression of many touch responsive genes.

From an ecological point of view, thigmomorphogenesis can be seen as a strategy for better resisting wind or water flow, as discussed in the article by Schoenlynck et al.

Stems of aerial plants, especially those of trees which can grow very tall, must combine mechanical stability and hydraulic conductivity. These two functions have been often seen as antagonistic, implying necessarily that trade-offs are made. There is actually no means to predict the way a tree will acclimate or will optimize the different functions of its wood by modifying the wood structure. In this research topic Badel et al. review acclimation of wood xylem anatomy, and its resulting mechanical and hydraulic functions to recurrent mechanical strains generally engendered by wind with a special focus on the construction costs and possible trade-off.

The research topic concludes with an article by Lopez et al. that explores the link between perception of mechanical signals and perception of gravity, and proposes new protocols for differentiating gravity-specific signal transduction and responses.

## Author contributions

All authors listed, have made substantial, direct and intellectual contribution to the work, and approved it for publication.

### Conflict of interest statement

The authors declare that the research was conducted in the absence of any commercial or financial relationships that could be construed as a potential conflict of interest.

